# Comparison of Different Recruitment Methods for Sexual and Reproductive Health Research: Social Media–Based Versus Conventional Methods

**DOI:** 10.2196/jmir.7048

**Published:** 2017-03-10

**Authors:** Yoko Motoki, Etsuko Miyagi, Masataka Taguri, Mikiko Asai-Sato, Takayuki Enomoto, John Dennis Wark, Suzanne Marie Garland

**Affiliations:** ^1^ Department of Obstetrics and Gynecology Yokohama City University School of Medicine Yokohama, Kanagawa Japan; ^2^ Department of Biostatistics Yokohama City University School of Medicine Yokohama, Kanagawa Japan; ^3^ Department of Obstetrics and Gynecology Niigata University Graduate School of Medical and Dental Science Niigata, Niigata Japan; ^4^ Department of Medicine and Bone and Mineral Medicine Royal Melbourne Hospital University of Melbourne Parkville, Victoria Australia; ^5^ Department of Microbiology and Infectious Diseases Royal Women’s Hospital Parkville, Victoria Australia; ^6^ Infection and Immunity Murdoch Childrens Research Institute Parkville, Victoria Australia; ^7^ Department of Obstetrics and Gynaecology University of Melbourne Parkville, Victoria Australia

**Keywords:** papillomavirus vaccines, reproductive health, sexual health, sexual behavior, Japan, uterine cervical neoplasms

## Abstract

**Background:**

Prior research about the sexual and reproductive health of young women has relied mostly on self-reported survey studies. Thus, participant recruitment using Web-based methods can improve sexual and reproductive health research about cervical cancer prevention. In our prior study, we reported that Facebook is a promising way to reach young women for sexual and reproductive health research. However, it remains unknown whether Web-based or other conventional recruitment methods (ie, face-to-face or flyer distribution) yield comparable survey responses from similar participants.

**Objective:**

We conducted a survey to determine whether there was a difference in the sexual and reproductive health survey responses of young Japanese women based on recruitment methods: social media–based and conventional methods.

**Methods:**

From July 2012 to March 2013 (9 months), we invited women of ages 16-35 years in Kanagawa, Japan, to complete a Web-based questionnaire. They were recruited through either a social media–based (social networking site, SNS, group) or by conventional methods (conventional group). All participants enrolled were required to fill out and submit their responses through a Web-based questionnaire about their sexual and reproductive health for cervical cancer prevention.

**Results:**

Of the 243 participants, 52.3% (127/243) were recruited by SNS, whereas 47.7% (116/243) were recruited by conventional methods. We found no differences between recruitment methods in responses to behaviors and attitudes to sexual and reproductive health survey, although more participants from the conventional group (15%, 14/95) chose not to answer the age of first intercourse compared with those from the SNS group (5.2%, 6/116; *P*=.03).

**Conclusions:**

No differences were found between recruitment methods in the responses of young Japanese women to a Web–based sexual and reproductive health survey.

## Introduction

Prior research about sexual and reproductive health of young women has largely relied on self-reported survey studies. These surveys are challenging because of their low response rates and the potential bias of their participants [1].

Low survey response rates are caused by attitudes of young adults toward health issues and their personal lifestyles. Young adults have less interest in health research than older adults, as they have fewer health problems in general and do not perceive themselves to be at risk. Young adults are also extremely mobile during their late teens and 20s because of college, new jobs, or becoming married. Consequently, targeted mailings are not always received. Moreover, computerized assisted telephone interviews are less optimal today, as fewer homes have landlines. Biases can also occur in self-reported sexual and reproductive health surveys and include non-respondent bias and social disability bias [1]. Direct interviews and self-administered questionnaires may be biased in settings such as clinics, schools, and workplaces, even if they are submitted anonymously [1]. This could be because the participants felt nervous or uncomfortable with the teachers or parents learning about their sexual behavior.

Thus, social media–based recruitment to surveys can potentially improve the low response rate and biases in survey-based sexual and reproductive health research [2]. In a prior study, we reported that social media such as Facebook is a cost-efficient and a promising way to reach a general population of young women for sexual and reproductive health research [3-9]. In addition, Facebook is one of the popular social networking sites (SNSs) worldwide. Unlike the other SNSs such as LINE, mixi, and LinkedIn, Facebook can provide demographic-targeted advertisement (eg, gender, age, or place of residence) using the cost-per-click option, a low-cost system to charge per number of user clicks or views [9]. We have used Facebook previously in research to recruit general populations of young women, as it is one of the most popular SNSs that young women use to communicate. However, little is known whether social media–based or conventional recruitment methods (ie, face-to-face or flyer distribution) yield comparable survey responses from similar participants [2,10].

We conducted a survey to determine whether there was a difference in the sexual and reproductive health survey responses of young Japanese women based on recruitment methods: social media–based and conventional methods.

## Methods

### Study Oversight

This study was a component of a pilot study for the Yokohama-Kanagawa Cervical Cancer Prevention Project [3]. This study protocol was approved by the Institutional Ethics Committee of the Yokohama City University School of Medicine. The detailed methodology of this study was previously reported by Miyagi et al [3].

### Targeted Participants

We recruited women aged 16-35 years in the Kanagawa Prefecture, Japan, with a population of 9 million, between July 2012 and March 2013 (9 months).

#### Recruitment Through Facebook

We defined participants recruited through Facebook as the “SNS group.” We recruited them by using a Facebook advertisement in the cost-per-click option.

#### Recruitment by Conventional Methods

We defined participants recruited through conventional methods as the “conventional group.” The conventional methods used included face-to-face recruitments in clinics and workplaces, and flyer distributions at schools, hospitals, and educational events, which accepted our concept of sexual and reproductive health research.

### Enrollment

All participants were invited to access the Yokohama-Kanagawa Cervical Cancer Prevention Project website, through which their personal information was obtained. Subsequently, they were sent a link via email, where they were asked to complete a Web-based questionnaire (SurveyMonkey; SurveyMonkey, Inc).

### Statistical Analysis

We asked the participants whether they felt embarrassed by the Web-based survey questions on a scale of 1-5 (1 = not embarrassing, 5 = extremely embarrassing). We also used a chi-square analysis of independence to compare the responses of the two different recruitment groups. We compared the scores between the two groups using Student’s *t* test. We analyzed the data by SPSS version 20 (IBM Corporation). A *P* value of < .05 was considered statistically significant.

## Results

### Attitudes to the Survey Between Recruitment Methods

During the 9-month study period, 394 participants expressed their interests and 264 consented to the study. Among the 243 who completed the Web-based questionnaire, the SNS group　(52.3%, 127/243) and the conventional group (47.7%, 116/243) had similar attitudes for all questions regarding their sexual behavior, except for the question regarding the age when they became sexually active. Among those already sexually active (86.8%, 211/243), more participants in the conventional group (15%, 14/95) denied answering the age of their sexual debut, compared with those in the SNS group (5.2%, 6/116) (*P*=.03; Table 1).

**Table 1:** Characteristics of participants recruited through social media–based and other conventional methods.

Characteristics		Total (n=243)				SNS group (n=127)				Conventional group (n=116)			
		No	Rate (%)	95% CI		No	Rate (%)	95% CI		No	Rate (%)	95% CI	*P*
**General**													
**Age Group**													
	16-19	14	5.76	(2.8-8.7)		4	3.15	(0.1-6.2)		10	8.62	(3.5-13.7)	.10
	20-29	122	50.21	(43.9-56.5)		65	51.18	(42.5-59.9)		57	49.14	(40.0-58.2)	
	30-35	107	44.03	(37.8-50.3)		58	45.67	(37.0-54.3)		49	42.24	(33.3-51.2)	
**District of residence**													
	Yokohama City	143	58.85	(52.7-65.0)		75	59.06	(50.5-67.6)		68	58.62	(49.7-67.6)	>.99
	Others	100	41.15	(35.0-47.3)		52	40.94	(32.4-49.5)		48	41.38	(32.4-50.3)	
**Educational level^a^**													
	< High school graduate	5	2.07	(0.3-3.9)		2	1.57	(0.0-3.7)		3	2.61	(0.0-5.5)	.88
	High school graduate	47	19.42	(14.4-24.4)		26	20.47	(13.5-27.5)		21	18.26	(11.2-25.3)	
	> High school graduate	190	78.51	(73.3-83.7)		99	77.95	(70.7-85.2)		91	79.13	(71.7-86.6)	
**Sexual and reproductive health **													
**Sexual experience**													
	No answer	1	0.41	(0.3-0.5)		0	0.00	NA		1	0.86	(0.8-0.9)	.05
	Never	31	12.76	(8.9-17.4)		11	8.66	(3.8-13.6)		20	17.24	(11.1-25.1)	
	Yes	211	86.83	(82.6-91.1)		116	91.34	(86.4-96.2)		95	81.90	(74.9-88.9)	
**Age at first intercourse^b^**													
	No answer	20	9.48	(5.5-13.4)		6	5.17	(1.1-9.2)		14	14.74	(7.6-21.9)	.03
	12-15	14	6.64	(3.3-10.0)		9	7.76	(2.9-12.6)		5	5.26	(0.8-9.8)	
	16-18	71	33.65	(27.3-40.0)		46	39.66	(30.8-48.6)		25	26.32	(17.5-35.2)	
	19-24	98	46.45	(39.7-53.2)		52	44.83	(35.8-53.9)		46	48.42	(38.4-58.5)	
	25-30	8	3.79	(1.2-6.4)		3	2.59	(0.0-5.5)		5	5.26	(0.8-9.8)	
**Condom use at first sex^b^**													
	No answer	2	0.95	(0.0-2.3)		1	0.86	(0.0-2.5)		1	1.05	(0.0-3.1)	>.99
	Yes	171	81.04	(75.8-86.3)		94	81.03	(73.9-88.2)		77	81.05	(73.2-88.9)	
	No	38	18.01	(12.8-23.2)		21	18.10	(11.1-25.1)		17	17.89	(10.2-25.6)	
**Number of sexual partner within 12 months^b^**													
	0-2	180	85.31	(80.5-90.1)		98	84.48	(77.9-91.1)		82	86.32	(79.4-93.2)	.85
	3+	18	8.53	(4.8-12.3)		12	10.34	(4.8-15.9)		6	6.32	(1.4-11.2)	
	No answer	13	6.16	(2.9-9.4)		6	5.17	(1.1-9.2)		7	7.37	(2.1-12.6)	
**Chlamydia awareness**													
	Yes	226	93.00	(89.8-96.2)		121	95.28	(91.6-99.0)		105	90.52	(85.2-95.8)	.21
	No	17	7.00	(3.8-10.2)		6	4.72	(1.0-8.4)		11	9.48	(4.2-14.8)	
**Positive test history for chlamydia among whom they knew chlamydia**													
	Yes	28	12.39	(8.1-16.7)		14	11.57	(5.9-17.3)		14	13.33	(6.8-19.8)	.84
	No	197	87.17	(82.8-91.5)		106	87.60	(81.7-93.5)		91	86.67	(80.2-93.2)	
	No answer	1	0.44	(0.0-1.3)		1	0.83	(0.0-2.4)		0	0.00	N.A.	
**Awareness of HPV vaccines^b^**													
	Yes	194	79.84	(74.8-84.9)		99	77.95	(70.7-85.2)		95	81.90	(74.9-88.9)	.52
	No/No answer	49	20.16	(15.1-25.2)		28	22.05	(14.8-29.3)		21	18.10	(11.1-25.1)	
**Self-reported cervical cancer screening status among whom their age** ≥ **20^c^**													
	Yes	156	68.42	(62.1-74.2)		85	69.67	(61.5-77.8)		71	66.98	(58.0-75.9)	.67
	No	45	19.74	(14.5-24.8)		20	16.39	(9.8-23.0)		25	23.58	(15.5-31.7)	
	No answer	27	11.84	(8.0-16.5)		17	13.93	(7.8-20.1)		10	9.43	(3.9-15.0)	
**Self-reported HPV vaccination status**													
	Yes	30	12.35	(8.2-16.5)		11	8.66	(3.8-13.6)		19	16.38	(9.6-23.1)	.08
	No	206	84.77	(80.3-89.3)		113	88.98	(83.5-94.4)		93	80.17	(72.9-87.4)	
	Don’t know/No answer	7	2.88	(0.8-5.0)		3	2.36	(0.0-5.0)		4	3.45	(0.1-6.8)	

^a^One participant did not answer this question and was excluded.

^b^Question only for participants who answered 'yes' to having had sexual experiences.

^c^One who answered “I don't know” was excluded. The Japanese government invites women aged 20 years and older to cervical cancer screening.

Among women who were sexually active, there was no significant statistical difference in the age of their sexual debut between the two groups (mean age, [SD]), SNS: 19.4 [2.8 years], Conventional: 18.9 [2.6 years], *P*=.16, by Student’s *t* test).

### Differences in Responses to the Survey Between the Recruitment Methods

**Figure 1:**
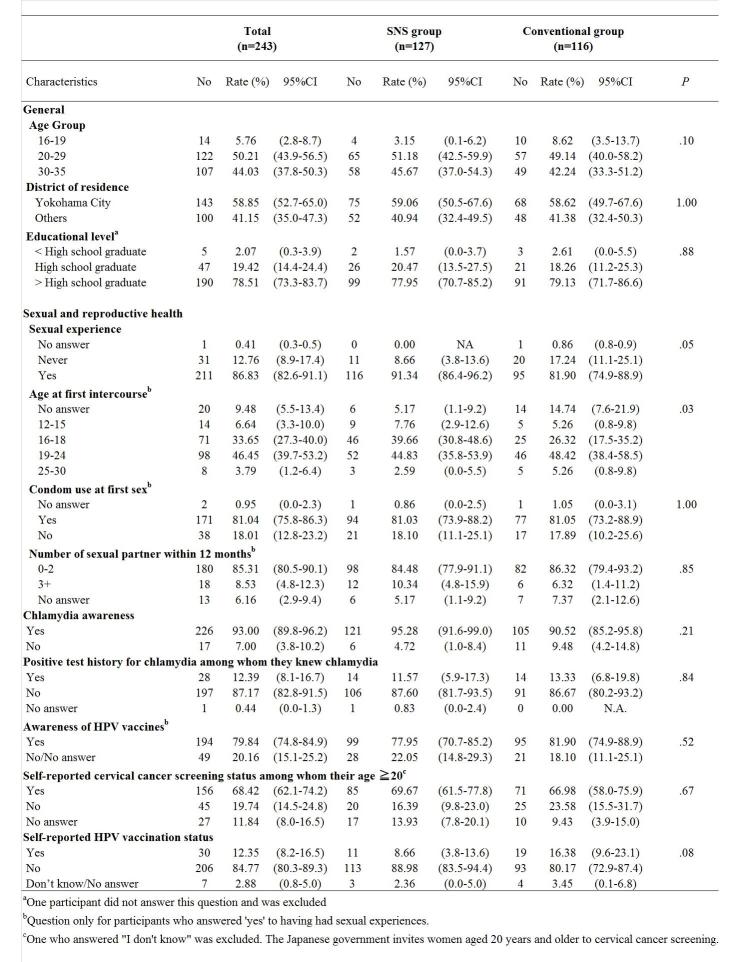
Mean embarrassing scores for Web-based questionnaires about sexual and reproductive behaviors among participants recruited through social media–based and other conventional methods.

There was no statistical difference between the two groups in their responses to questions (embarrassing scores) about the age when they became sexually active, number of sexual partners, regular condom use, sexually transmitted infection, and active use of oral contraceptives (Figure 1).

## Discussion

### Principal Findings

Through this study, we found that Japanese women have similar responses to questions regarding sexual behavior and health, regardless of the way in which they were recruited.

We conducted this study in a similar manner to a prior Australian study by Fenner et al [9], but which did not incorporate conventional methods. We added the conventional methods because we were concerned that there were fewer social media users among Japanese women (72% in their teens and 64% in their 20s) than among Australian women (83% of 16-29-year-old women in 2010) [11].

Today in Japan (as of April 2016), 25 million people access their Facebook accounts monthly and 64% of them daily [12]. The use of smartphone has increased and encourages the frequent use of SNSs [13].

In our study, among those who were sexually active, more participants in the conventional group did not respond to the question regarding the age of sexual debut. We do not consider that this difference means a conflict to similarity between recruitment methods. There are three reasons. First, there was no demographic difference between sexually active and nonactive (data not shown). Second, means and SDs of ages of sexual debut were similar between the two groups. Third, attitudes toward the Web-based survey were the same, irrespective of the recruitment method used (Figure 1). Therefore, we hypothesize that there might be a difference caused by anxiety about anonymity with our conventional methods of recruitment. This hypothesis should be verified by further research.

We can therefore assert that SNSs are promising and efficient tools for participant recruitment for sexual and reproductive health research.

### Limitation

A limitation of this study was that we were able to recruit the SNS group as a randomized sample but not the conventional group. Some participants might have felt nervous about the possibility that their friends, teachers, or coworkers would learn about their sexual behavior, especially among participants in the conventional group. This might have resulted in a difference in the response rate to the question about age of sexual debut.

### Conclusions

SNSs have made revolutionary changes in medical research. However, with respect to recruitment of study participants, these changes may be too large or rapid to permit validation of any differences between conventional and SNS-based recruitment methods. In this study, we confirmed similarities between these methods of recruitment for participation in a Web-based sexual and reproductive health survey. Cervical cancer prevention in Japan is declining. This is exemplified by low Papanicolaou cervical cytology screening rates which have resulted in increased cervical cancer mortality and incidence rates among younger Japanese women [14]. Moreover, the suspension of the human papillomavirus (HPV) vaccination promotion by the Japanese government in June 2013 has also decreased the coverage rate for the HPV vaccine from approximately 70% to almost zero [15]. Evidence-based measures must be taken to overcome the cervical cancer epidemic in Japan. This study confirmed that a nationwide SNS-based survey targeting young Japanese women would be a realistic way to spread information and awareness about cervical cancer prevention.

## Abbreviations

HPV: human papillomavirus
SD: standard deviation
SNS: social networking site
